# Sciatica—A Neuritis, Rarely a Neuralgia-Illustrative Cases1Read before the Central New York Medical Association at its annual meeting, held at Rochester, October 20, 1896.

**Published:** 1897-01

**Authors:** E. B. Angell

**Affiliations:** Rochester, N. Y. 293 Alexander Street


### SCIATICA—A NEURITIS, RARELY A NEURALGIA—ILLUSTRATIVE CASES.[1]

By E. B. ANGELL, M. D., Rochester, N. Y.

SCIATICA is a term so easily applied to any severe, recurring pain along the thigh, that rarely is sufficient examination made by the general practitioner to determine the exact nature of the disorder. With the mistaken idea that it is a neuralgia or rheumatism, objective signs are not sought for; the tale of the sufferer, with his undoubted expression of pain, and his stiff, limping gait being sufficient to establish a careless diagnosis and too frequently a fruitless treatment.

When, however, objective indications are searched for, it will be readily found that in the majority of cases the neuralgic pain, as such, proceeds from a true inflammation of the nerve, a neuritis, of which the pain is the most marked symptom. Neuralgia, in its present restricted sense, may be defined as a neurosis, characterised by spontaneous pain, due to functional disturbance or irritation of a nerve rather than to organic change in its tissues. The term neuralgia, then, should be restricted to those cases of nerve pain, idiopathic in character, or symptomatic of irritation from without, but not applied to pain due to organic changes of a nerve. In neuritis or inflammation of a nerve, we have definite objective signs of alteration in its structure, although pain may be the chief symptom, and decidedly of most importance to the victim. Nerve inflammation in general has certain pathognomonic symptoms, and if severe, results in organic changes of a motor and sensory character, readily recognised, very distinct from the functional disturbances due to neuralgia alone.

Any series of cases of sciatica, if carefully studied, will reveal a symptomatology which can better be explained as the result of a true neural inflammation, the appreciation of which will go far toward the establishment of satisfactory treatment.

In the early stage of the trouble, acute pain along the course of the nerve trunk, shooting, boring, burning in character, or a dull heavy ache, worse at night, is the essential symptom. So far it is a true neuralgia. When, however, the nerve is inflamed, if pressure is made along its course it will be found sensitive; decidedly so at certain points—the sciatic notch, the middle of the thigh, the popliteal space and at the ankle. Even though the ten-

1. Read before the Central New York Medical Association at its annual meeting, held at Rochester, October 20, 1896.

derness along the thigh is not marked, slight stretching of the nerve will readily elicit pain. This may be accomplished by seating the patient on a chair with the knee of the affected leg at a little more than a right angle. If now the finger is pressed into the popliteal space, so as to increase the tension of the nerve, a pain is produced along the course of the nerve in the back of the thigh, or above the sciatic notch if the seat of inflammation is within the pelvis. Perverted sensation, such as tingling, formication, numbness, burning and the like, may be felt in the area of its peripheral distribution, owing to pressure upon the nerve bundles within the swollen sheath. In severe cases, through destruction of the nerve filaments, there may be discovered irregular areas of anesthesia, flabby, atrophied muscles from motor disturbances, with alteration in electrical irritability which only in extreme cases amounts to a distinct reaction of degeneration. Vaso-motor changes are manifested rarely through herpetic eruption, exfoliation of the epidermis, or edema of the leg. Seldom does the inflammation extend upward to the plexus or cord, although permanent damage to these structures has resulted from sciatica.

How different the picture of a true neuralgia! While a neuralgia may give rise to vaso-motor disturbances, it is but temporary —as short-lived as the pain itself—and rarely more than a flushing of the blood-vessels of the corresponding area.

Except the nerve fibers be damaged, we do not find motor or sensory changes, or alteration of electrical reactions in our examination. But in a large proportion of these cases of so-called sciatica, careful investigation will develop the characteristic pain upon pressure or tension, some sensory alteration, muscular wasting and other signs indicative of irritation, pressure or destruction of nerve fibers. Essentially, sciatica is an inflammation of the nerve sheath, whence it may extend and affect the nerve framework, interstitial neuritis, or in the most severe cases attack the nerve fibers themselves, the parenchymatous type.

Furthermore, one curious fact is strongly corroborative of this distinction. In general, neuralgia attacks women more frequently than men, but in sciatica the reverse is true, the proportion being in the ratio of four males to one female.

I am well aware that some cases of sciatica are dependent upon causes without the nerve itself, such as rectal and other tumors, pelvic inflammation, bone disease and the like, while certain toxic states of the blood may give rise to neuralgic pain as well.

Indeed, in some cases a rectal examination is necessary to clear up an obscure cause. But neuralgia alone is a relatively rare form of the disease, and he who seeks a diagnosis from that standpoint will frequently meet with disappointment in his therapeutics.

The treatment of sciatica in general, then, corresponds to the therapeutics applicable to a neuritis. In the acute stage of the inflammation, absolute rest or immobility of the nerve is of prime importance. If the attack be mild, this may be secured by simple rest in bed, but in the severer forms—and it is the stubborn cases I am discussing—it is imperative to apply a splint to the leg, slightly flexed, in order to protect the irritated nerve. I know of no measure of more importance, yet rarely have I found it employed by the general practitioner.

Rest from pain may demand opiates, of which, injection of morphia is best. But, until compelled to that course, I much prefer the deep injection of cocaine in the neighborhood of the tender points, being careful to avoid wounding the nerve itself. This drug not only acts as a local analgesic, but antagonises the congested condition as well.

Mercury is generally regarded as serviceable in inflammation and may prove of advantage. It is to be administered in the form of blue pill or calomel. At least it will counteract in a measure the constipating effect of opiates, as well as promote elimination. In severe inflammatory cases, a hot, generous poultice along the posterior aspect of the thigh will give relief, though in the purely neuralgic type, cold will be more efficient.

Counter-irritation should be used early. The actual cautery, lightly applied along the course of the nerve, is of most value, while flying blisters or acupuncture may be tried. Galvanism is worse than useless in the acute stage, but will be found of real value when the disorder has become chronic, in which stage static electricity has proved itself of signal service. If muscular atrophy has resulted as a sequela, the faradic current, with slow interruptions, will do much toward restoring tone and strength to the wasted tissues. In those cases depending upon lithemia or toxic states of the blood, the free exhibition of salicylin, the salicylates and alkalies, has proved of decided value. Doubtless, they are the most serviceable drugs we have in this condition.

In stubborn chronic cases, nerve stretching may be of decided value, as it proved in a recent case of mine. It is rarely necessary to cut down upon the nerve itself, as sufficient force usually may be brought to bear upon the nerve trunk, by manipulation of the thigh. With the patient well under ether, the leg extended is gradually but forcibly flexed upon the trunk by the surgeon, until any nerve adhesions are thoroughly loosened. This must not be done in any timid manner, or it will fail of accomplishing its purpose. Indeed, ligaments may be torn or muscular fibers ruptured, but the nerve must be subjected to sufficient force to insure success, it being borne in mind that the nerve will sustain a breaking strain of thirty or forty pounds.

I have limited my suggestions in therapeutics to the special measures applicable to the treatment of a neuritis, presupposing the needlessness of calling attention to the value of generous nourishment and the necessity of securing thorough elimination.

Permit me briefly, before closing, to call attention to three typical and obstinate cases, all of them referred to me by general practitioners after vain attempts to cure by the usual methods.

CASE I.—*Chronic Sciatic Neuritis.*—Mr. F. G., retired merchant, aged 60 years; family history negative; a personal history of lithemic dyspepsia for many years. The year previous, an attack of lumbago with sequent left sciatica. Treatment gave amelioration but not relief. At the time he came to me the disease had become chronic. He complained of burning sensation, as though hot water was being poured along the thigh; a tingling and numbness in the foot, and difficulty in straightening up from a bending posture. Pressure elicited soreness along the nerve trunk, especially marked at the sciatic notch, under the knee and at the ankle. The skin was dry and scaly. The muscles of the upper part of the thigh, especially the gluteal, were wasted, the circumference of the left thigh being two centimeters less than that of the right at the gluteal fold. The electrical reactions were altered, though no true reaction of degeneration was elicited. Sensation was dulled over area of distribution of the left sciatic nerve.

Distinctly the trouble was due to neuritis, and a treatment suitable to that condition in the chronic stage was instituted. Galvanism of the thigh, with faradic stimulation of the weakened muscles, was applied every other day. Salicylates and alkalies were administered, while the dyspeptic trouble was corrected. The result was full recovery in three weeks, except a slight lameness from the muscular weakness, which steadily lessened in time without further attention.

CASE II.—*Acute Form of Neuritis.*—Mr. M. A., *æt.* 42, traveling salesman; family and personal history negative. Eight months previous to consulting me, a sharp attack of "rheumatism,"

so-called, across the back and in the abdomen, lasting two weeks. Then it affected the left sciatic nerve, and had been very severe ever since. Exposure the undoubted cause. Slight improvement had been followed by exacerbations of increasing severity. Present attack very distressing, with sharp, shooting, burning pain throughout course of nerve, greatly increased by movement or even by standing. Sensations of "pins and needles," with a burning pain in top of left foot. Foot was dusky and slightly swollen. Sensation to touch decidedly diminished over area of nerve distribution, somewhat increased to temperature. Painful points upon pressure along the nerve trunk, most marked in the popliteal space. Muscles of left leg less firm than those of right, but wasting only slight. Kneejerks exaggerated, decidedly so on left side.

Diagnosis, acute sciatic neuritis. Treatment, absolute rest in bed, leg on a long splint; cocaine injections, and morphia when pain uncontrolled by the former drug; light, frequent counter-irritation with the paquelin cautery over the line of nerve in thigh. Medication, a blue pill daily, with saline cathartics and salicylates freely. The acute pain was relieved, but there developed an obstinate chronic condition of dull ache throughout the leg and burning sensation in the calf and paresthesia in the foot, indicative of pressure upon the nerve filaments from a swollen sheath. In June last, under ether, the nerve was thoroughly stretched by Dr. Kempe, through manipulation. Adhesions were broken up, with incidental rupture of muscle fibers and, doubtless, some tearing of ligamentous tissue. The leg was again put in a splint, as the pain was naturally decidedly worse at first. But gradual improvement was established, until today, some nine months since the operation, he is fully recovered and actively engaged in his occupation.

CASE III.—*Neuralgic Type.*—Mr. Conrad S., *æt.* 49, a farmer, German by descent; family and personal history good. Three years before, an attack of so-called sciatica, which wore itself out uninfluenced by drugs. A second attack two years later. At the time I saw him he had suffered for ten weeks excruciating agony, with muscular cramps, hardly controlled by heavy doses of morphia. Pain worse at night, causing marked insomnia. There was always a dull, aching, frequently burning and shooting pains, with distinct periodic exacerbations, affecting chiefly, but not exclusively, the left sciatic area. There was considerable gastralgia at times. Frequent and painful cramps of the left leg muscles were present. Upon examination, I found no sensory impairment in the sciatic area; no sensitiveness of the nerve trunk upon pressure; no painful points along the nerve. The electrical reactions were normal, the muscles unimpaired, except through the general wasting. Indeed, there was lacking any evidence of a neuritis. On the other hand, there was a decided manifestation of the lithemic diathesis, confirmed by urinary analysis.

Here, then, was a true neuralgia, dependent upon a toxic blood state, a marked contrast to the cases above recorded.

With appropriate anti-lithemic treatment the diathesis was remedied and the sciatic neuralgia speedily relieved, local treatment consisting solely of counter-irritation by the galvano-cautery upon two or three occasions.

In my remarks upon this oftentimes obstinate malady I have endeavored merely to determine the distinction that should be made between sciatic neuritis and the neuralgic form, by no means venturing to discuss the causes, course and management of the disease. If it shall lead to a closer study of a common ailment and a clearer comprehension of its management my purpose shall have been fully accomplished.

293 Alexander Street.

